# Baicalin promotes β-1,3-glucan exposure in *Candida albicans* and enhances macrophage response

**DOI:** 10.3389/fcimb.2024.1487173

**Published:** 2024-12-09

**Authors:** Yiyuan Pan, Zhaoling Shi, Yadong Wang, Feng Chen, Yue Yang, Kelong Ma, Wenqian Li

**Affiliations:** ^1^ College of Integrated Chinese and Western Medicine (College of Life Science), Anhui University of Chinese Medicine, Hefei, China; ^2^ Institute of Integrated Chinese and Western Medicine, Anhui Academy of Chinese Medicine, Hefei, China; ^3^ Anhui Provincial Key Laboratory of Chinese Medicinal Formula, Hefei, Anhui, China

**Keywords:** Candida albicans, baicalin, β-1,3-glucan, macrophage, transcriptome

## Abstract

Among the diverse fungal opportunistic pathogens, *Candida albicans* garners significant attention due to its wide range of infections and high frequency of occurrence. The emergence of resistance and the limited number of antifungals drives the need to develop novel antifungal drugs. Although the natural product baicalin has been shown to trigger apoptosis in *C. albicans* in previous experiments, its influence on cell wall (CW) structure along with immune recognition remains elusive. In this work, baicalin showed a significant killing effect against *C. albicans* SC5314. Moreover, CW destruction, characterized by β-1,3-glucan unmasking and chitin deposition, was observed as a consequence of the treatment with baicalin. The RNA sequencing analysis revealed that treatment with baicalin resulted in eight hundred forty-two differentially expressed genes (DEGs). Sixty-five genes, such as GSC1, ENG1, CHS3, GWT1, and MKC1, were associated with CW organization or biogenesis. Baicalin-pretreated *C. albicans* SC5314 was phagocytosed more efficiently by RAW264.7 macrophages, accompanied by increased TNF-α and IL-1β production. Accordingly, it is hypothesized that baicalin could stimulate β-1,3-glucan unmasking by governing CW-associated gene expression in *C. albicans* SC5314, which contributes to macrophage recognition and clearance.

## 1 Introduction

*Candida albicans*, a main opportunistic fungal pathogen, usually colonizes the oral, gastrointestinal, and genital tracts asymptomatically. The fungus can induce infection when perturbation of endogenous microbiota, host immune system, or both occurs. Mucosal or even systemic infections caused by *C. albicans* are two medically important subtypes. In particular, systemic candidiasis is always accompanied by high mortality, such as candidemia (Lopes and Lionakis, 2022). Considering the emergence of resistance, restrictions on use, and the limited number available, novel antifungals must be developed (Boyce, 2023).

The absence of cell wall (CW) structures in human host cells renders them a prominent target for developing novel antifungal therapies (Cortés et al., 2019). Although the CW of *C. albicans* is generally stable, it exhibits a flexible structure. For instance, the outer layer is abundant in proteins with high levels of mannose, and the inner layer consists of β-1,3/1,6-glucan and chitin. Owing to the central role of β-1,3-glucan in the CW structure, it has emerged as the most promising antifungal target (Ibe and Munro, 2021). The pathogen-associated molecular pattern (PAMP) β-1,3-glucan is masked by superficial mannans to conceal immunogenic epitopes. Upon the CW remodeling, the exposed β-1,3-glucan can be detected by pattern recognition receptors (PRR), including dectin-1, that is located on the innate immune cell surface, thereby facilitating phagocytosis and clearance of *C. albicans* (Bojang et al., 2021). Both environmental signals [acidic pH (Sherrington et al., 2017) and iron (Tripathi et al., 2020)] and antifungals [caspofungin (Walker and Munro, 2020; Wagner et al., 2023), micafungin (Guirao-Abad et al., 2018), amphotericin B (Pradhan et al., 2019), and fluconazole (Pradhan et al., 2019)] cause β-1,3-glucan unmasking. This, in turn, could enhance the innate immune response to *C. albicans* by macrophage and produce a stronger pro-inflammatory cytokine response. Limited studies have reported the role of traditional Chinese herbal medicine-derived active compounds that reinforce *C. albicans-*produced β-1,3-glucan in inducing the innate immune response.

The active constituents of medicinal plants are often used as a potential source for the development of antifungals (Curto et al., 2021; Heard et al., 2021). From this context, berberine hydrochloride (Huang et al., 2021) and sodium houttuyfonate (Da et al., 2019; Wang et al., 2024) derived from traditional Chinese herbal medicine can induce CW remodeling, thereby facilitating β-1,3-glucan moiety unmasking. Baicalin is a major *Scutellariae radix-*extracted flavonoid that exhibits significant bioactive properties. These attributes include antimicrobial activity, anti-inflammatory capabilities, and immunomodulatory activities (Huang et al., 2019). Recent investigations have revealed that baicalin increases *C. albicans* apoptosis rate, highlighting its role in combating fungal infections (Yang et al., 2014; Wang et al., 2015). Nevertheless, the precise inhibitory mechanisms of baicalin on the *C. albicans* β-1,3-glucan *in vivo* exposure and colonization remain unclear.

This study explored the underlying mechanism of baicalin in inducing β-glucan unmasking, enhancing macrophage immune response, and clearing *C. albicans*. Transcriptome analysis was harnessed to delve into the molecular mechanisms modulated by baicalin against *C. albicans*. These discoveries offer both theoretical foundations and experimental evidence to support the case for developing baicalin as a novel approach to the treatment of antifungal infections.

## 2 Materials and methods

### 2.1 Cell culture

Wild-type *C. albicans* strain SC5314 was provided by Professor Yuanying Jiang, stored at -80°C and activated in liquid yeast extract–peptone–dextrose (YPD) medium (Hope bio-Technology, China). Resuscitated SC5314 was inoculated to the solid YPD medium (Hope bio-Technology, China) by the plate scribing separation method and cultured at 37°C. Then, a selected single colony was amplified to the logarithmic phase of growth at 37°C, and 200 rpm overnight for subsequent experiments.

The RAW264.7 mouse macrophages acquired from the National Collection of Authenticated Cell Cultures were grown in Dulbecco’s Modified Eagle Medium (DMEM) medium (SparkJade, China) at 5% CO_2_ and at 37 °C. The medium contained 10% fetal bovine serum (FBS) (Anhui Kangyuan Biotechnology, China) and was supplied with 1% penicillin and streptomycin (PS) (Biosharp, China).

### 2.2 Susceptibility assay of fungi to drugs

Minimum inhibitory concentration (MIC) values of baicalin or fluconazole (both from Yuanye Biotechnology, China) against *C. albicans* were tested in microdilution plates at 37°C and examined visually after 24 h according to the recommendations in the Clinical and Laboratory Standards Institute (CLSI) M27-A3 document (Wang et al., 2015; Montoya et al., 2020). Both *Candida parapsilosis* ATCC 22019 and *Candida krusei* ATCC 6258 were used as quality control (QC) strains to confirm the accuracy and reproducibility in antifungal susceptibility testing (Guirao-Abad et al., 2018; Shields et al., 2018). Baicalin was serially diluted in 1.953–1000 μg/mL by Roswell Park Memorial Institute (RPMI)-1640 medium (Gibco, USA) and co-incubated with *C. albicans* SC5314 at a final concentration of 1 × 10^3^ CFU/mL. Fluconazole was tested at concentrations of 0.0625–8 μg/mL. The MIC endpoint was defined as the lowest concentration of the drug that results in a 50% reduction in fungal growth compared to the growth control (Holtappels et al., 2017; Shields et al., 2018; Brennan-Krohn et al., 2021).

### 2.3 Fungal viability

Using the Cell Counting Kit-8 (CCK-8), the dehydrogenase activity within the mitochondria of *C. albicans* SC5314 was determined to characterize cell viability (Jin et al., 2021). Briefly, 5 × 10^4^ CFU/mL of *C. albicans* SC5314 cells were dispensed into 96-well plates at 100 μL/well. Subsequently, these cells were exposed to 100 μL of different baicalin concentrations at 37°C. This enables the investigation of the concentration-dependent effects of baicalin on the cells. Afterward, 20 μL of CCK-8 reagent (Biosharp, China) was introduced into each well, followed by a 30-min incubation at 37°C. Thereafter, a multi-well spectrophotometric plate reader was deployed to quantitatively determine the optical density (OD) at 450 nm.

### 2.4 Spot assay

To study the drug effect on of *C. albicans* growth, a slight modification was made according to a previously outlined protocol (Wang et al., 2018). Fungal cells in the logarithmic phase were resuspended by RPMI-1640 medium and 10-fold diluted to create five final concentrations of 2 × 10^1^ – 2 × 10^5^ CFU/mL. The above concentrations of *C. albicans* SC5314 were co-incubated with multiple concentrations of baicalin or fluconazole in a ratio of 1:1 at 37°C. Fluconazole-treated group was used as the positive drug control group. After spotting 5 μL of the mixed culture solution onto YPD solid plates, the growth differences and clonogenic potential were observed following 24-h incubation at 37°C.

### 2.5 Time-kill curve

A time-kill curve was used to examine the killing kinetics of the baicalin or fluconazole against *C. albicans*. The experimental procedures were performed based on earlier described methodologies (Cantón et al., 2004; Elgammal et al., 2024), with appropriate modifications to suit our objectives. Logarithmic phase *C. albicans* cells were subjected to dilution in RPMI 1640 medium until reaching a final concentration of 2 × 10^3^ CFU/mL and then incubated with either baicalin or fluconazole at 37°C. Equal volumes of samples were extracted at the pre-determined time intervals, diluted, and seeded onto YPD agar plates to determine the number of viable cells.

### 2.6 Transmission electron microscopy analysis

The growth of *C. albicans* SC5314 at a final concentration of 2.5 × 10⁴ CFU/mL was investigated in RPMI 1640 medium, both in the presence and absence of baicalin or fluconazole, for 24 h at 37°C. The transmission electron microscopy (TEM) experiments were conducted based on the methodologies outlined in previous literature (Grigor’eva et al., 2020; Xie et al., 2022; Bing et al., 2024) with the incorporation of modest optimizations. The *C. albicans* samples were fixed with 2.5% glutaraldehyde and then by 1% osmium tetraoxide. Afterward, the samples were exposed to dehydration through an ethanol series and then embedded with an epon-araldite mixture. The embedded blocks were sliced into ultrathin sections of 70 nm in thickness, and then the slices were captured and supported on copper grids. A sequential staining process was then performed as follows, initially with 1% uranyl acetate, followed by Reynolds’ lead citrate (a mixture of lead nitrate and sodium citrate) for several minutes each. The TEM (Hitachi HT7700, Japan) was employed to capture images of stained sections.

### 2.7 Yeast cell surface staining, flow cytometry, and confocal microscopy analysis

The drug treatment procedure for *C. albicans* is outlined in Section 2.6. The cell surface staining was based on previous literature (Guirao-Abad et al., 2018; Wagner et al., 2023) and followed the manufacturer’s instructions. Both fluconazole- and caspofungin-treated groups were used as the positive drug control groups. For total chitin detection, the fungal cells collected by centrifugation were stained by 10 μg/mL of calcofluor white (Sigma-Aldrich, the United States) for 30 min with gentle shaking. The β-1,3-glucan monoclonal antibody (Biosupplies, Australia) and its corresponding Cyanine3-labeled secondary antibody (Abbkine, the United States) were utilized to evaluate exposed β-1,3-glucan. A confocal laser scanning microscope (Olympus FV3000, Japan) or flow cytometer (BD FACSCelesta™, the United States) was employed to examine the stained cells. Image J and FlowJo were used to analyze microscope photographs and flow cytometry data.

### 2.8 RNA extraction and sequencing

A 2.5 × 10^4^ CFU/mL of *C. albicans* strain SC5314 was co-cultured with 500 μg/mL baicalin in RPMI-1640 medium that contained 10% FBS. The control group was not supplemented with baicalin. There were three biological replicates in both the baicalin-treated and the control groups. Total RNA extraction was conducted through an RNA extraction kit (Majorbio, China) and the 5300 Bioanalyser (Agilent, the United States) was used for analyzing its quality. Only high-quality RNA samples meeting the criteria (amount > 1 μg, RIN ≥ 6.5, OD260/230 ≥ 2.0, OD260/280 = 1.8–2.2, and 28S: 18S ≥ 1.0) were utilized for RNA sequencing (RNA-seq) library construction. The library was generated following the Illumina^®^ Stranded mRNA Prep Ligation kit (Illumina, the United States). Using the Illumina NovaSeq 6000 high-throughput sequencer, the paired-end RNA-seq library sequencing was performed (PE library, 2 × 150 bp read length). The procedure for RNA extraction and sequencing is illustrated in **Supplementary Figure S1**.

### 2.9 Transcriptomic analysis

The fastp tool (Chen et al., 2018) was employed to conduct statistical evaluation and quality control on the raw paired-end reads. Then, the resulting clean reads were subjected to an individual mapping to the reference genome of *C. albicans* SC5314 (GCF_000182965.3) by HISAT2 (Kim et al., 2015) software to obtain mapped reads for subsequent analysis. The StringTie (Pertea et al., 2015) was applied to assemble the mapped reads for each sample.

Quantification and calculation of gene expression abundances were performed using RSEM (Li and Dewey, 2011) based on the approach of transcripts per million (TPM) reads. Using DESeq2 (Love et al., 2014) with parameters |log_2_foldchange| > 1 alongside adjusted P < 0.05, differentially expressed genes (DEGs) were identified between the control and baicalin groups. Furthermore, utilizing the Database for Annotation, Visualization and Integrated Discovery (DAVID), DEGs can be categorized through Kyoto Encyclopedia of Genes and Genomes (KEGG) pathway enrichment and Gene Ontology (GO) annotations on the basis of the biological processes (BP) they participate in, the cellular components (CC) they constitute, and the molecular functions (MF) they perform. A workflow of transcriptomic analysis is shown in **Supplementary Figure S1**.

Raw and processed data for transcriptome analysis was uploaded to the GEO database with accession number GSE249820.

### 2.10 Quantitative real-time PCR

The treatment procedures for *C. albicans* were the same as those outlined in Section 2.8, “RNA extraction and sequencing”, while the handling of RAW264.7 macrophages was detailed in Section 2.11, “Macrophage-mediated phagocytosis of *C. albicans*”. Following total RNA isolation from either *C. albicans* or RAW264.7 by yeast RNA kit (SparkJade, China) or FastPure cell/tissue total RNA isolation kit (Vazyme, China), respectively, we assessed RNA concentration and its quality through the NanoDrop™ One microvolume spectrophotometer (Thermo Fisher Scientific, the United States). Next, the extracted RNA was employed to synthesize complementary DNA (cDNA) via a cDNA synthesis kit (YEASEN, China). **Tables 1**, **2** list sequence-specific primers designed for quantitative real-time PCR (qRT-PCR). The qRT-PCR amplification system was prepared according to the qPCR SYBR green master mix kit (YEASEN, China) and performed on the LightCycler^®^ 96 instrument (Roche, Switzerland). The qPCR cycling parameters were set as follows: denaturation at 95°C for 2 min, annealing at 60°C for 10 s, and extension at 60°C for 30 s, with a total of 40 cycles. The internal reference genes ACT1 (for *C. albicans* SC5314) and Actb (for *Mus musculus*) were detected for data normalization. Here, the 2^-ΔΔCt^ method was employed as a relative quantification strategy for data analysis. All the operations were performed in an RNase-free environment.

**Table 1:** qRT-PCR primers (for *Candida albicans* SC5314).

Gene	Forward primer (5’ to 3’)	Reverse primer (5’ to 3’)
ACT1	TGCTGAACGTATGCAAAAGG	TGAACAATGGATGGACCAGA
GSC1	ATCAACAACCACTTGCTTCG	CCCATTCTCTAGGCACGA
GSL1	TATGTCATTCAACTCGCCTTCCTTG	AATCAGATATTGCGGCACCTAACG
GSL2	GGCATCACTTCGCTCCCAAAC	AGCTTGTTCTAACTTGTCGTTCTCC
BGL2	CTCGCAACTGTTCTTACTTCAGTTG	TGACGTCTTTACAAGTACCGTCATC
ENG1	AAGCCGACTCCAGTTCAAGTTCAG	AACTGTTGGTGGAGCGTTGGTATC
GWT1	CGGACGAACTATTGCCGAGAAAAC	TCATTGACGTGCCCCATGTTTCC
PHR1	GGTTTGGTTCTGGTTGATGG	AGCAGCAGTTCCTGGACATT
PHR2	CTCCTCCATTTCCAGAACCA	CGTCTGAATCAACCTTGTCG
MNN12	CACTCACCAGACACGGCAATG	TCTTCTTCTTGGTGTTGCTGACC
CHS3	AGATTCTATTGCCACCACCGATTAC	CACAGTCAAGTCCGACATCATATCC
CHS4	TGCCACTGAATTAACTGCTGCTG	AATGACCTAATGCTGCTGCTTGAG
CDC42	CCGTCATTTCTCCTGCTTCGTTTG	TTATTGGCACACCGGGACAATGG
CST20	GCGTTTGGCGGTGAGAATAATGC	GTGGAGGCGGCGGAGGTG
STE11	CCTGCTCATGATCGCCATTCTAC	ACCAAATTCTCCATCTTCCTCACC
HST7	CAAGCGGTAGCCTAAGGAGTTCTG	TAGGTGGCGGGCGTTGTCTC
CEK1	GCTCAGGCTCAGGCTCAGG	TGAATGAAACTTGACGAGGGGAAG
YPD1	GACCATGGACGAGGATGAAGAAGG	TGACCCGACGATGACAATTTCTCC
SSK1	AGCAGCATCAGCATCAGCATCAG	ACTTGTTCTGTGGTGTGGTCAACG
SSK2	TCACCAACGACACCCGCAATAAG	GACGACCTGGCGATTTCTCTCTTG
PBS2	TACCAACACCACCAACTCCCTCAG	TGCTGCTGCTGCTGCTTTCG
HOG1	TGATCCAACCGATGAGCCTGTTTG	ACCCTCAGTTTCATTTGCCACACC
RHO1	TGAGACAACAACAACAACAACCAG	CCAAGTAATCAGCAGCACCAATTC
PKC1	ATGCCAAGCCGTTACCACCTAAAC	CCACTGACGCTGGTCGCAAAG
BCK1	ACACGAACGACGAGCAACCATC	ACTTTGCGACGAGCCTCATTGG
MKK2	AGGACTCAAGTTGTCAACGTCTCC	GATTCGTCAGCAGAGCCCTGTTG
MKC1	TCCCACAGAGCCCAGAACTATGTC	CGGGTTGGCGTCAGGGAAAAG

**Table 2:** qRT-PCR primers (for *Mus musculus*).

Gene	Forward primer (5’ to 3’)	Reverse primer (5’ to 3’)
TNF-α	AAGCCTGTAGCCCACGTCGTA	AAGGTACAACCCATCGGCTGG
IL-1β	CAACCAACAAGTGATATTCTCCATG	GATCCACACTCTCCAGCTGCA
IL-10	CTTACTGACTGGCATGAGGATCA	GCAGCTCTAGGAGCATGTGG
Actb (β-actin)	ATCTGGCACCACACCTTCTACAATG	CACGCTCGGTCAGGATCTTCATG

### 2.11 Macrophage-mediated phagocytosis of *C. albicans*

The RAW264.7 macrophages were seeded at 6 × 10^5^ cells/mL into 35 mm confocal dishes (NEST, China) and cultured overnight. A 2.5 × 10^4^ CFU/mL of *C. albicans* SC5314 was pretreated with or without 1000 μg/mL of baicalin at 37°C for 24 h. Subsequently, harvested *C. albicans* cells were co-incubated with RAW264.7 cells at a 3:1 multiplicity of infection (MOI; *C. albicans*: macrophage) in DMEM for 1 or 3 h. After incubation, the culture medium was eliminated, and fungus-macrophage co-cultures were gently rinsed with PBS three times. Nonphagocytosed *C. albicans* SC5314 cells were labeled with 10 μg/mL of calcofluor white (CFW), while phagocytosed *C. albicans* cells remained unstained. Afterward, excess CFW dye was washed away with PBS, followed by fixing the cells with 4% paraformaldehyde. The sample image was acquired by the Olympus FV3000 microscope imaging system. The formula described before was applied to calculate the phagocytic index (Tripathi et al., 2020). Total RNA was extracted from fungus-macrophage co-cultures, and the relative IL-1β/10 and TNF-α mRNA expression from macrophages was detected and calculated.

### 2.12 Statistical analysis

Statistical significance was conducted utilizing Student’s *t*-test (two-tailed), with each experiment being performed with three biological replicates. The data were statistically analyzed, and the corresponding figures were generated via GraphPad Prism Version 9.5.0.

## 3 Results

### 3.1 Baicalin antifungal activity

**Figure 1:**
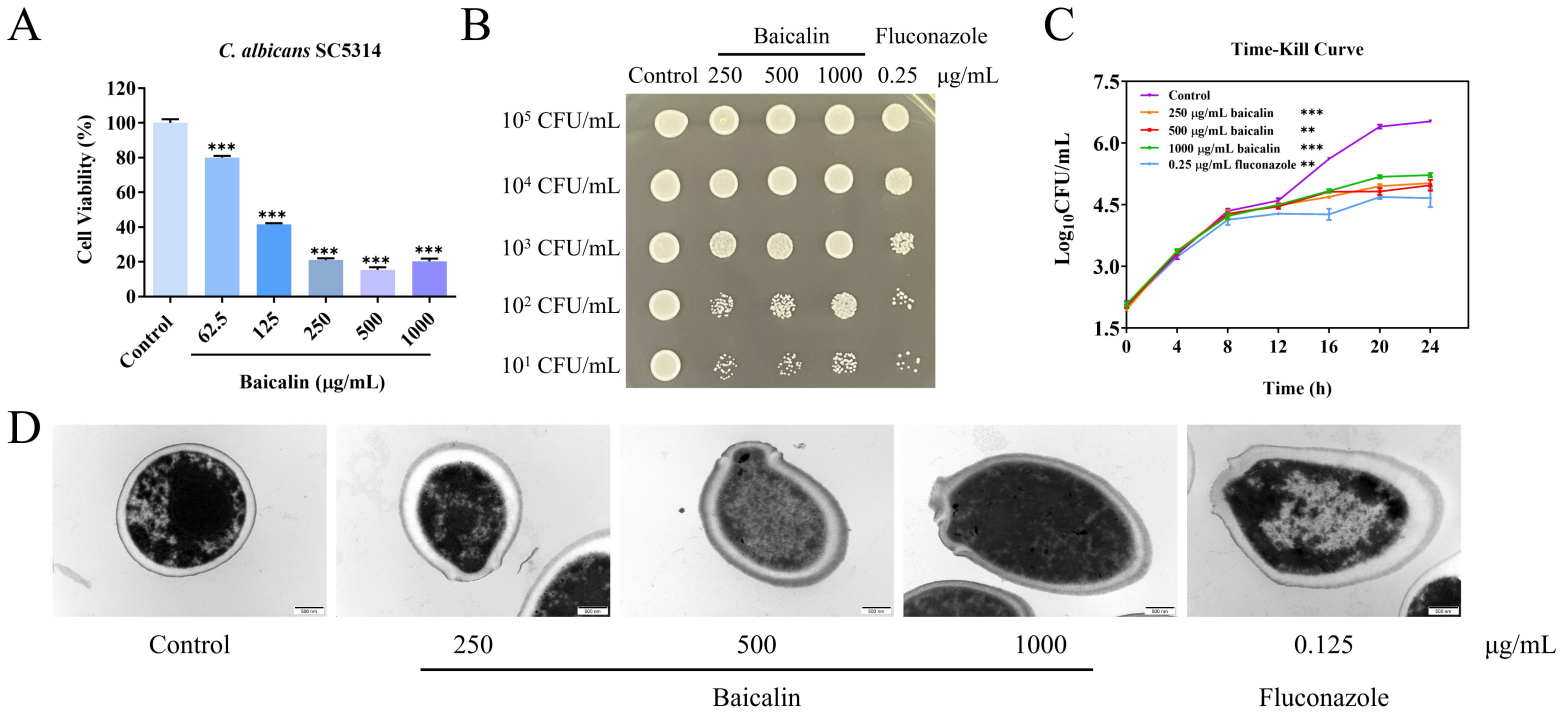
Baicalin antifungal properties against *C*. *albicans* strain SC5314. **(A)** Cellular viability of *C*. *albicans* subjected to diverse concentrations of baicalin. **(B)** Spot assay: Detecting growth inhibition of *C*. *albicans* SC5314 on plates. **(C)** The time-kill curves: *C*. *albicans* SC5314 growth kinetics. **(D)** Ultrastructure of *C*. *albicans* SC5314 under TEM; scale bar = 500 nm. Data represent mean values ± standard deviations (SD). The mean values and standard deviations reported were based on three independent experiments. Two-tailed unpaired Student’s *t*-test was used for statistical comparisons. **p < 0.01 and ***p < 0.001 vs. the control group.

The MIC of baicalin on *C. albicans* SC5314 yeast cell strain and fluconazole were 500 and 0.25 μg/mL, respectively. **Figure 1A** depicts that *C. albicans* SC5314 cell viability was 15% when treated with 500 μg/mL (MIC) and 21% when treated with 250 μg/mL (1/2 MIC). When the initial inoculation concentration of *C. albicans* SC5314 was less than or equal to 10^2^ CFU/mL, the number of colonies formed on plates after baicalin treatment exhibited a marked decrease in contrast to the control group (**Figure 1B**). In the time-dependent killing curve, the killing activity of baicalin in the first 12 h was similar to that of the control. However, after 12 h, *C. albicans* SC5314 growth was significantly impeded by baicalin above 250 μg/mL (**Figure 1C**). The maintenance of CW integrity is a critical virulence determinant in *C. albicans* SC5314 pathogenicity (Lima et al., 2019). Upon exposing *C. albicans* SC5314 cells to baicalin, alterations in the cellular ultrastructure were imaged with TEM. The structural outline of the CW appeared blurred, with partial thinning observed, and in some instances, exhibited signs of breakage and disintegration (**Figure 1D**). Collectively, baicalin can suppress *C. albicans* SC5314 virulence by interfering with its CW integrity.

### 3.2 Baicalin induces β-glucan exposure and increases the chitin content at the *C. albicans* cell surface

**Figure 2:**
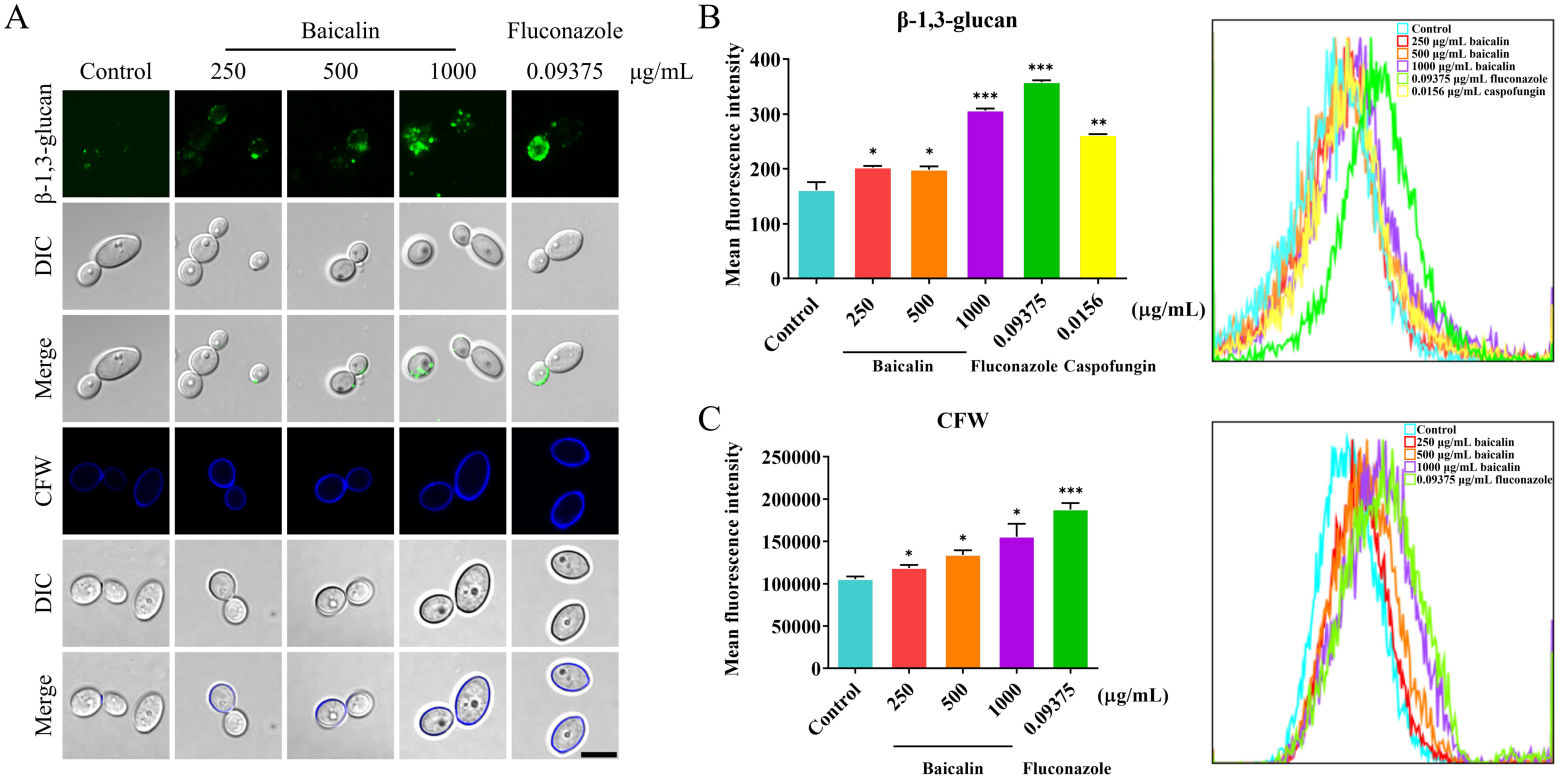
Changes in β-1,3-glucan exposure and total chitin within the cellular wall in response to treatment with baicalin in *C*. *albicans* SC5314. **(A)** Fluorescence microscopy: β-1,3-glucan exposure (green) and total chitin (blue) on *C*. *albicans* SC5314 wild-type cell surface. Flow cytometry: Quantitative measurement of MFI for **(B)** β-glucan unmasking and **(C)** total chitin; Scale bar = 5 µm. Data represent means ± standard error of the mean (SEM) from at least three independent experiments. Statistical comparisons between groups were performed using Student’s *t*-test (two-tailed, unpaired): *p < 0.05, **p < 0.01, and ***p < 0.001 vs. control group.

The mechanism underlying the baicalin-induced CW remodeling remains unclear at present. This study thoroughly examined the antifungal mechanism of baicalin by focusing on β-glucan exposure, which is a main PAMP in the CW. The microscopy examination revealed that the CW surface exhibited more staining for β-1,3-glucan and chitin in the baicalin-treated group compared to the control group (**Figure 2A**). Cell surface fluorescence was quantitatively assessed through flow cytometry by determining the mean fluorescence intensity (MFI). The results confirmed that the MFI of β-1,3-glucan was enhanced upon baicalin treatment (**Figure 2B**). The same as β-1,3-glucan, the MFI of CFW-labeled total chitin was increased with baicalin treatment (**Figure 2C**). This indicated that baicalin induces β-glucan unmasking and chitin deposition in *C. albicans* SC5314.

### 3.3 Identification of candidate genes related to CW remodeling in *C. albicans* in response to baicalin by global transcriptomic analysis

**Figure 3:**
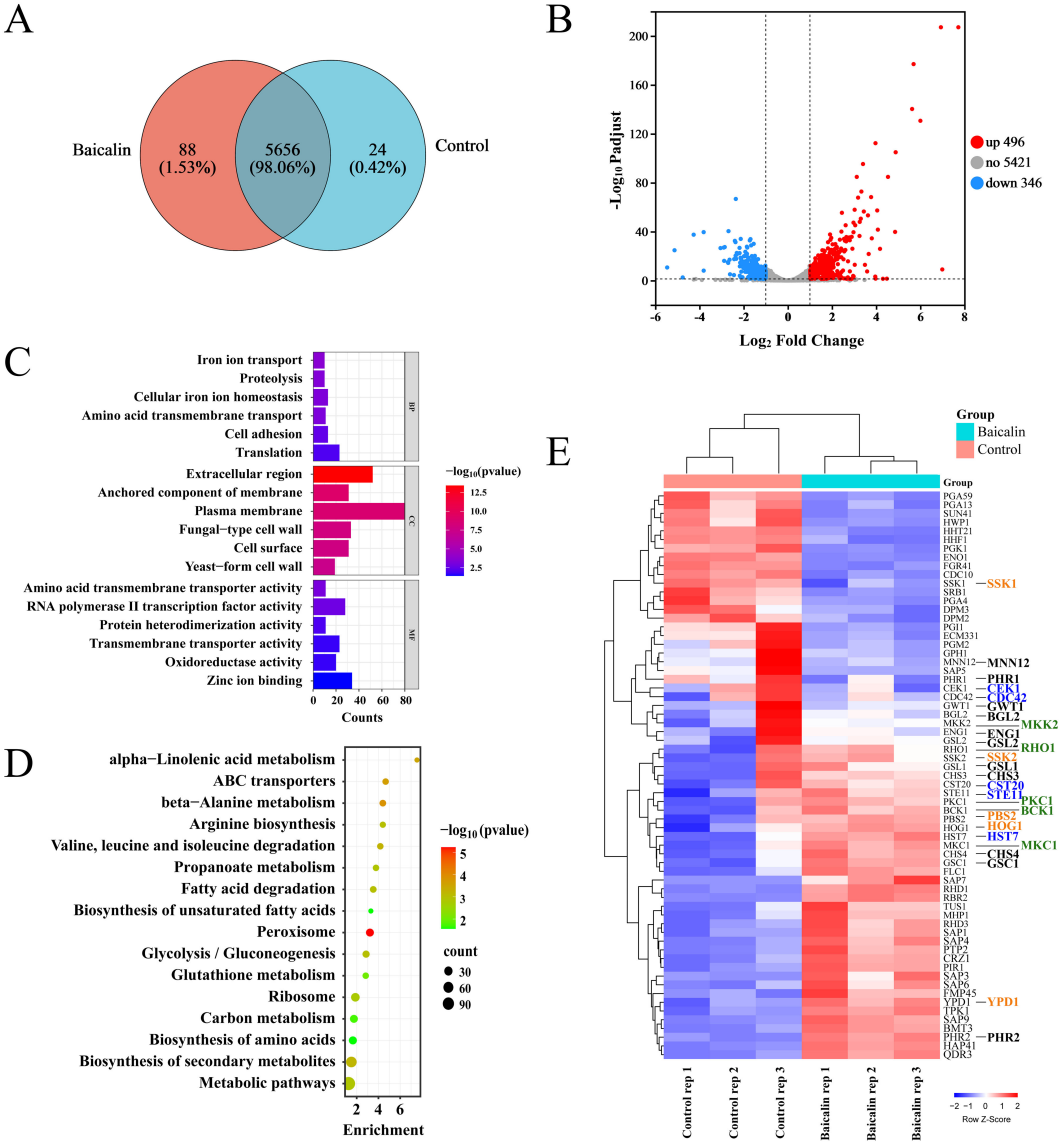
Transcriptomic analysis of the *C. albicans* SC5314 in response to treatment with baicalin for CW remodeling. **(A)** Correlation between transcripts of the baicalin-treated and control groups. **(B)** Volcano plot displaying differential expression statistics of genes: Up-regulated (red dots), down-regulated (blue dots), and non-significantly DEGs (gray dots). **(C)** GO enrichment analysis of DEGs categorized genes based on their involvement in BP, CC, and MF. **(D)** Analysis of KEGG pathways for DEGs identifying enriched pathways. **(E)** Heatmap of gene expression clustering analysis for genes that participated in CW organization or biogenesis in the baicalin-treated or control group. Genes associated with the Cek1 pathway are denoted by the color blue, those linked to the Mkc1 pathway are colored green, genes participated in the Hog1 pathway are displayed in yellow font, and genes potentially associated with β-1,3-glucan exposure are marked in black.

Triplicate samples from each of the baicalin-treated and control groups were subjected to RNA-seq using the Illumina NovaSeq 6000 high-throughput transcriptome sequencing platform. A total of 5768 transcripts were annotated in the sequencing samples from both groups, of which 5656 transcripts were shared between the two groups (**Figure 3A**). Out of the 842 identified DEGs, 496 were up-regulated and 346 were down-regulated in the baicalin-treated groups compared to the control group (**Figure 3B**). The GO annotation and functional enrichment analysis results showcased that DEGs were primarily enriched in CC, such as fungal CW and plasma membrane, as well as BP, including translation, cell adhesion, and iron ion transport (**Figure 3C**; **Supplementary Table S1**). The KEGG pathway enrichment analysis results manifested that DEGs exhibited a main enrichment in carbon metabolism, glycolysis, and peroxisome pathways (**Figure 3D**; **Supplementary Table S2**). To investigate the mechanism behind β-1,3-glucan exposure, GO annotation was employed to screen out 50 genes related to the CW organization or biogenesis in *C. albicans* (**Figure 3E**; **Supplementary Table S3**). Additionally, the 15 genes associated with signal transduction pathways (Cek1, Hog1, and Mkc1 pathways) within *C. albicans* that regulate β-1,3-glucan unmasking (Chen et al., 2022) were of particular interest (**Figure 3E**; **Supplementary Table S3**). The products encoded by genes, including MNN12, CHS3/4, PHR1/2, BGL2, GSC1, GSL1/2, ENG1, and GWT1, were involved in CW organization and might directly or indirectly regulate β-1,3-glucan unmasking (**Figure 3E**; **Supplementary Table S3**).

### 3.4 Gene expression involved in β-1,3-glucan unmasking after baicalin treatment

**Figure 4:**
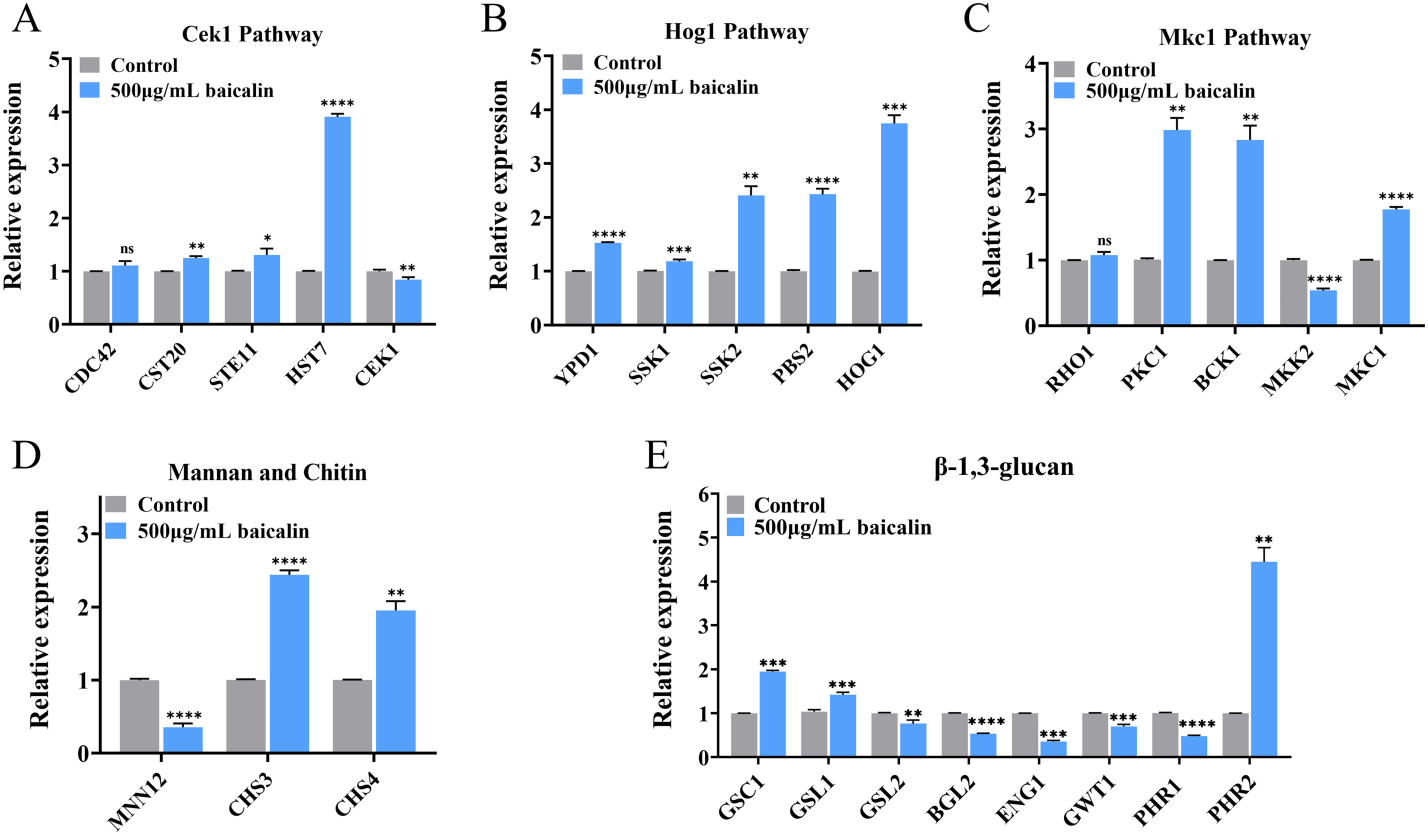
Relative mRNA expressions of genes mediating β-1,3-glucan exposure after baicalin treatment. Relative expressions of genes in the MAPK pathways: the **(A)** Cek1, **(B)** Hog1, and **(C)** Mkc1 pathways and genes participated in CW component synthesis and degradation: **(D)** mannan, chitin, and **(E)** β-1,3-glucan. Data represent mean values ± SD of three independent experiments. Comparisons were conducted with a two-tailed unpaired Student’s *t*-test: *p <0.05, **p < 0.01, ***p < 0.001, and ****p < 0.0001.

The mRNA expression of 15 genes in the three MAPK pathways governing β-1,3-glucan exposure was confirmed by qRT-PCR. After treatment with baicalin, the expression of most genes within these pathways was up-regulated (**Figures 4A–C**). Moreover, α-1,3-mannosyltransferase (encoded by MNN12) was down-regulated after baicalin treatment (**Figure 4D**). The loss of the mannan outer layer of the CW may cause the inner layer β-1,3-glucan to unmask. Chitin synthases, particularly those encoded by CHS3/4, exhibited increased expression levels (**Figure 4D**), which was consistent with the elevated expression of total chitin observed by laser scanning confocal microscopy (**Figure 2A**) and flow cytometry (**Figure 2C**).

In *C. albicans* SC5314, the genes GSC1 and GSL1/2 encode for β-1,3-glucan synthases. An evident GSC1 and GSL1 overexpression was detected, whereas GSL2 expression displayed a significant decrease (**Figure 4E**). This observed distinct gene expression pattern indicated a possible β-1,3-glucan exposure induction through corresponding synthesis pathways. Relative expressions of glucan 1,3-β-glucosidase (encoded by BGL2) and glucan endo-1,3-β-D-glucosidase 1 (encoded by ENG1) was down-regulated, which may increase exposure by reducing the degradation of β-1,3-glucan (**Figure 4E**). Inhibition of GPI biosynthesis caused the unmasking of *C. albicans* β-glucan layer (Huang et al., 2020; Chen et al., 2022). GPI-anchored wall transfer protein 1 (encoded by GWT1) was down-regulated (**Figure 4E**). The pH-responsive proteins 1/2 (encoded by PHR1/2, respectively) have 1,3-β-glucanosyltransferase activity, in which the PHR1 expression level decreased, whereas the PHR2 expression level increased (**Figure 4E**).

### 3.5 Baicalin pretreatment promotes *C. albicans* phagocytosis and macrophage cytokine production

**Figure 5:**
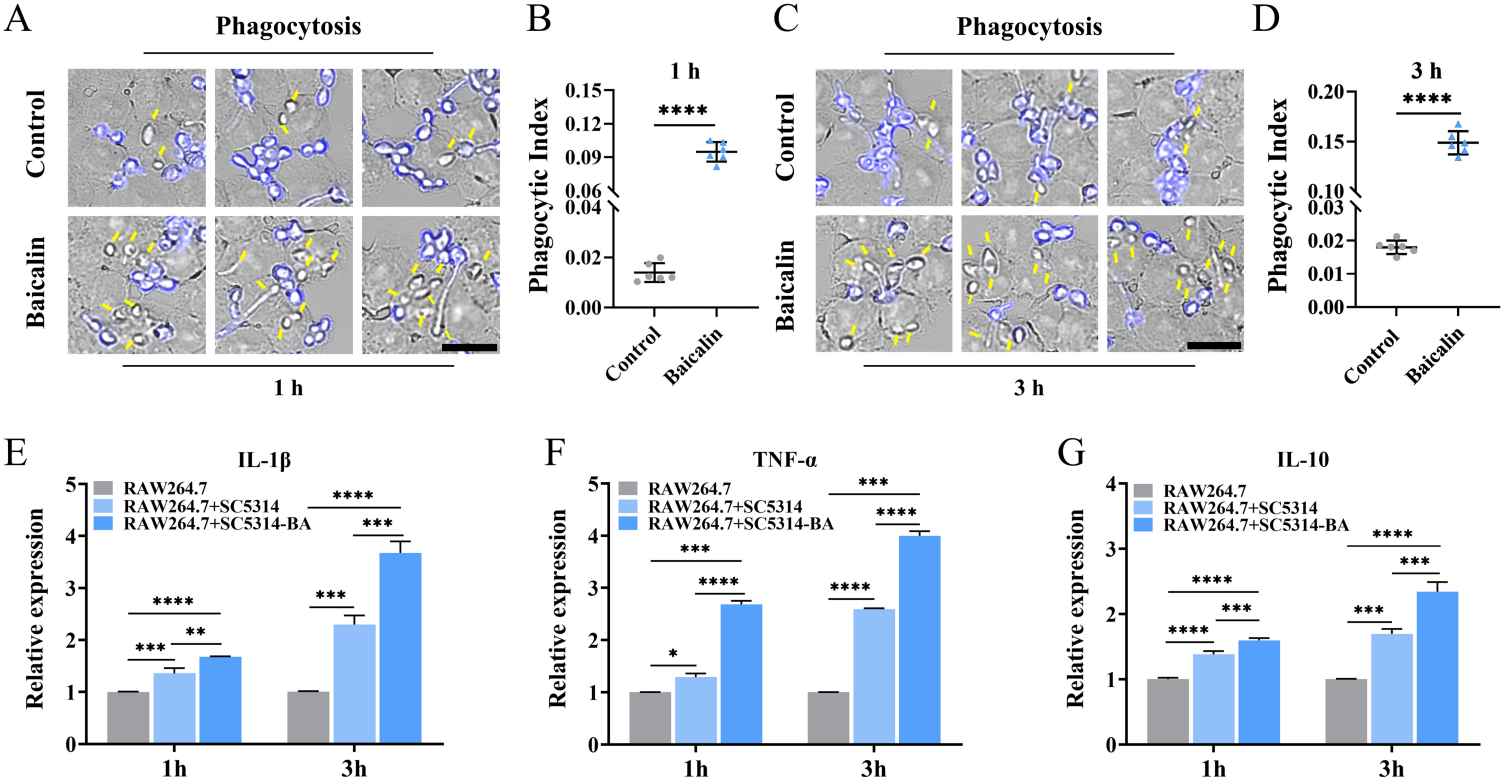
C. *albicans* pretreatment with baicalin enhances the immune response of the host. RAW264.7 infection with *C*. *albicans* SC5314 pretreatment with baicalin (1000 μg/mL) at an MOI of 3 for **(A)** 1 or **(C)** 3 h. External blue-labeled *C*. *albicans* cells with CFW and unstained phagocytosed *C*. *albicans* cells, as indicated by yellow arrows. Micrographs showing statistics of the phagocytic index for **(B)** 1 and **(D)** 3h. Relative mRNA expressions of inflammatory cytokines **(E)** IL-1β, **(F)** TNF-α, and **(G)** IL-10 responses from RAW264.7. There were three experimental groups: a macrophage-only (RAW264.7), macrophages and *C*. *albicans* SC5314 co-incubation group (RAW264.7 + SC5314), and RAW264.7 against *C. albicans* SC5314 group pretreated with 1000 μg/mL baicalin (RAW264.7 + SC5314 - BA) at an MOI of 3 for 1 or 3 h. Mean ± SD for three independent experiments. Two-tailed unpaired Student’s *t*-test was used for statistical analysis: *p < 0.05, **p < 0.01, ***p < 0.001, and ****p < 0.0001; Scale bar = 15 μm.

Aiming to investigate the host immune response against *C. albicans* pretreated with baicalin, RAW264.7 macrophages were exposed to *C. albicans* pretreated with baicalein or not. The phagocytosis of macrophages towards *C. albicans* cells, particularly those exhibiting increased β-1,3-glucan unmasking post-baicalin treatment (**Figure 2A**), exhibited a significant enhancement. This enhancement was quantified as a 6.8-fold increase after 1 h (**Figures 5A, B**) and an 8.3-fold increase after 3 h (**Figures 5C, D**) in the phagocytic index. Furthermore, infection of *C. albicans* SC5314 caused macrophages to secrete more cytokines. The *C. albicans* SC5314 pretreated with baicalin stimulated significantly higher pro-inflammatory cytokine (IL-1β and TNF-α) and anti-inflammatory cytokine (IL-10) levels from RAW264.7 in comparison to *C. albicans* SC5314 unpretreated with baicalin (**Figures 5E–G**).

## 4 Discussion

Currently, the primary categories of commonly used antifungals in clinical practice are polyenes (e.g., amphotericin B), azoles (e.g., fluconazole), and echinocandins (e.g., caspofungin). Prolonged and excessive use of antifungals has led to the emergence of drug resistance in *Candida* species (Lee et al., 2021). Among them, amphotericin B targets the fungal cell membrane ergosterol, which also causes toxic effects on the host cell membrane due to the similarities between ergosterol and cholesterol (Anderson et al., 2014). Therefore, the clinical use of amphotericin B is not recommended. The azole fluconazole has been the most commonly used drug class in clinical practice, but the emergence of azole resistance has also become a problem (Pristov and Ghannoum, 2019). Echinocandins demonstrate antifungal activity by destroying CWs that are completely absent in mammalian cells. Recently, the widespread clinical use of echinocandins has resulted in the development of resistance (Trovato et al., 2021). Accordingly, novel drugs or therapeutic interventions must be developed.

In this study, baicalin demonstrated significant inhibition of *C. albicans* SC5314 cell viability. In addition, baicalin significantly inhibited colony formation and had a killing effect on *C. albicans* SC5314 after 12 h of co-incubation. Notably, the CW integrity of *C. albicans* SC5314 was destroyed after baicalin treatment. Previous research believed that the CW is an exceptionally suitable target for drugs to combat fungal infections. This is due to several pieces of evidence: First, the CW molecules are absent in human cells; Second, the CW components are essential for fungal survival or virulence (Gow et al., 2017); Third, conserved innate immune PRR could recognize essential microbial components in fungal CWs (Hopke et al., 2018). This study and previous ones have discovered the *in vitro* antifungal activity of baicalin. Abnormalities in immune recognition and clearance functions cause opportunistic pathogenic fungus infections. Accordingly, it is crucial to ascertain whether baicalin can exert antifungal activity from the perspective of the interaction between *C. albicans* and innate immunity.

Because fungi mask PAMPs to elude the immune system, particularly β-1,3-glucan (Hopke et al., 2018), the focus is on exploring the alterations in CW structure, particularly β-1,3-glucan unmasking post-baicalin treatment. Baicalin could activate CW remodeling of *C. albicans* SC5314, including β-1,3-glucan exposure and total chitin increase. The CW, a vital fungal cell surface structure, determines cell shape, viability, and interactions with the environment. Moreover, CW construction is an intricate process involving over a thousand genes responsible for a network of biosynthesis, metabolism, and signal transduction (Gow et al., 2017). Therefore, RNA-seq methods were used to analyze the global transcriptome of *C. albicans* SC5314 to explore the potential molecular mechanism of baicalin-provoked β-1,3-glucan exposure.

MAPK pathway activation, including Cek1, Mkc1, and Hog1 MAPK, facilitates β-1,3-glucan unmasking in *C. albicans*. The gene expression in the Cek1 pathway was up-regulated as a whole, but the downstream gene CEK1 was slightly down-regulated in qRT-PCR analysis. Some studies have suggested that β-1,3-glucan exposure is attributed to CEK1 gene activation (Chen et al., 2019a, 2019b), while others believe it is CEK1 disruption (Galán-Díez et al., 2010). Gene expression in the Hog1 pathway was significantly up-regulated; Hog1 pathway activation is crucial in β-1,3-glucan exposure (Hopke et al., 2016; Correia et al., 2019; Huang et al., 2021). The MKC1 deletion reduced β-1,3-glucan unmasking of caspofungin-treated *C. albicans* (Wagner et al., 2023). Therefore, it is hypothesized that MKC1 and up-regulation of gene expression in the Mkc1 pathway also promote β-1,3-glucan exposure. Herein, only the transcriptional regulation of the three MAPK pathways was explored, and it is still necessary to explore gene expression at the translation and post-translational levels in the future.

In addition to the signaling pathways, genes associated with CW organization or biogenesis could directly or indirectly affect β-1,3-glucan unmasking. Most of the downstream genes regulated by the above pathways are CW-related genes. Flow cytometry and microscopy imaging results manifested that increased total chitin accompanied by β-1,3-glucan unmasking, which aligns with previous studies. This chitin deposition in the inner layer of CW could disrupt the CW structure, thereby unmasking β-1,3-glucan (Chen et al., 2022). Our study elucidated that both CHS3/4 were overexpressed. Chitin synthase 3, encoded by CHS3, is the key chitin synthase enzyme liable for chitin deposition within the CW (Bulawa et al., 1995; Mio et al., 1996; Walker et al., 2008). The initiation of the repair mechanism following CW damage triggers its remodeling, resulting in increased chitin content and a corresponding CW thickening. Fungal cells could maintain their CW integrity through such adaptive mechanisms (Walker et al., 2008; Lee et al., 2012; Ibe and Munro, 2021; Wagner et al., 2023). Moreover, deleting genes encoding superficial mannans unmasked β-1,3-glucan in several fungal pathogens enhanced the innate immune cell response to fungi via interaction with dectin-1 (Bain et al., 2014; Gow et al., 2017). The decrease in MNN12 expression might also contribute to the reduced surface layer of mannans and β-1,3-glucan unmasking in the inner layer (Chen et al., 2022). In addition, β-1,3-glucan synthase gene GSL2 was decreased, while GSC1 and GSL1 were increased. A decrease in β-1,3-glucan synthases could expose β-1,3-glucan through CW remodeling mechanisms, such as echinocandins (Hoehamer et al., 2010). Similarly, it is speculated that increased β-1,3-glucan synthases will also trigger β-1,3-glucan exposure through CW integrity disruption. Conversely, trimming of β-1,3-glucan by ENG1 (Yang et al., 2022) could promote β-1,3-glucan masking to prevent dectin-1 detection. In response to baicalin exposure, the expression of β-1,3-glucosidase ENG1 and BGL2 decreased to facilitate β-1,3-glucan exposure.

Besides mannoproteins, chitin, and β glucan, which make up the CW, other factors govern β-1,3-glucan exposure. The gepinacin-inhibited GWT1, an essential acyltransferase participating in GPI anchor synthesis, led to the unmasking of CW β-glucan, further stimulating the pro-inflammatory cytokine response in macrophages (McLellan et al., 2012). Therefore, the down-regulation of GWT1 may also promote the β-1,3-glucan unmasking. Furthermore, PHR1/2 contributing to the cross-linking of β-1,3/6-glucan (Fonzi, 1999; Calderon et al., 2010) were differentially expressed.

To conclude, we identified the possible molecular mechanism of baicalin-induced β-1,3-glucan exposure using RNA-seq and qPCR, including the expression changes of genes associated with the MAPK signaling pathway and CW organization or biogenesis at the transcriptional level. The mechanism and analysis mentioned above are still relatively broad and lack a focused and in-depth analysis. In the future, one of these specific regulatory mechanisms can be further explored by introducing molecular biology techniques (such as gene knockout strains), pathway-related inhibitors, and animal experiments.

The innate immune system serves as the first defense line against *C. albicans*-induced infection. The β-1,3-glucan is an important immunorecognition epitope for macrophages. The *C. albicans* have evolved various strategies to mask β-1,3-glucan to evade phagocytic recognition (d’Enfert et al., 2021). In this study, baicalin-pretreated *C. albicans* SC5314 showed an increased rate of macrophage phagocytosis and stimulated macrophages to release more inflammatory cytokines.

The mechanisms of compounds derived from traditional Chinese medicine against *C. albicans* infections mainly focus on targeting cell membrane (e.g., magnolol), CW components (e.g., sodium houttuyfonate), mitochondria (e.g., berberine, curcumin, and silibinin), and virulence factors (e.g., honokiol and cinnamaldehyde) (Liu et al., 2017). Previous studies on the mechanisms of baicalin against *C. albicans* have conducted in-depth explorations, including inhibiting the activity of mitochondrial enzymes, reducing proliferation, promoting apoptosis, and impeding the biofilm formation of *C. albicans* (Yang et al., 2014; Wang et al., 2015). Herein, baicalin may induce β-1,3-glucan unmasking by disturbing gene expression that contributes to CW organization or biogenesis in *C. albicans* SC5314 and enhances macrophage recognition and phagocytosis by β-glucan receptor dectitn-1.

Although the antifungal resistance of existing drugs in clinical practice cannot be ignored (Fisher et al., 2022), only a few antifungal drugs, such as caspofungin and isavuconazole, have been approved in recent decades (Cui et al., 2022; Roy et al., 2023). This research helps us understand the antifungal mechanism of baicalin and provides new insights into the clinical application of *C. albicans* infections, focusing on fungal and host interactions. Accordingly, further *in vitro* and *in vivo* experiments should be conducted on clinical isolates of *C. albicans* to provide more experimental evidence for the clinical use of baicalin either as monotherapy or in combination therapy.

## Data Availability

Raw and processed data for gene expression analysis was deposited in the GEO database under accession number GSE249820. Other data contained within this article are available from the article/**Supplementary Material**, further inquiries can be directed to the corresponding authors.

## References

[B1] Anderson T. M. Clay M. C. Cioffi A. G. Diaz K. A. Hisao G. S. Tuttle M. D.. (2014). Amphotericin forms an extramembranous and fungicidal sterol sponge. Nat. Chem. Biol. 10, 400–406. doi: 10.1038/nchembio.1496 24681535 PMC3992202

[B2] Bain J. M. Louw J. Lewis L. E. Okai B. Walls C. A. Ballou E. R.. (2014). Candida albicans hypha formation and mannan masking of β-glucan inhibit macrophage phagosome maturation. MBio 5, e01874. doi: 10.1128/mBio.01874-14 25467440 PMC4324242

[B3] Bing J. Guan Z. Zheng T. Ennis C. L. Nobile C. J. Chen C.. (2024). Rapid evolution of an adaptive multicellular morphology of Candida auris during systemic infection. Nat. Commun. 15, 2381. doi: 10.1038/s41467-024-46786-8 38493178 PMC10944540

[B4] Bojang E. Ghuman H. Kumwenda P. Hall R. A. (2021). Immune sensing of candida albicans. J. fungi (Basel Switzerland) 7 (2), 119. doi: 10.3390/jof7020119 PMC7914548 33562068

[B5] Boyce K. J. (2023). The microevolution of antifungal drug resistance in pathogenic fungi. Microorganisms 11 (11), 2757. doi: 10.3390/microorganisms11112757 38004768 PMC10673521

[B6] Brennan-Krohn T. Friar L. Ditelberg S. Kirby J. E. (2021). Evaluation of the Synergistic Activity of Antibacterial and Antifungal Drugs against Candida auris Using an Inkjet Printer-Assisted Method. Antimicrob. Agents Chemother. 65, e0026821. doi: 10.1128/AAC.00268-21 34252295 PMC8448112

[B7] Bulawa C. E. Miller D. W. Henry L. K. Becker J. M. (1995). Attenuated virulence of chitin-deficient mutants of Candida albicans. Proc. Natl. Acad. Sci. U. S. A. 92, 10570–10574. doi: 10.1073/pnas.92.23.10570 7479842 PMC40653

[B8] Calderon J. Zavrel M. Ragni E. Fonzi W. A. Rupp S. Popolo L. (2010). PHR1, a pH-regulated gene of Candida albicans encoding a glucan-remodelling enzyme, is required for adhesion and invasion. Microbiology 156, 2484–2494. doi: 10.1099/mic.0.038000-0 20430812

[B9] Cantón E. Pemán J. Gobernado M. Viudes A. Espinel-Ingroff A. (2004). Patterns of amphotericin B killing kinetics against seven Candida species. Antimicrob. Agents Chemother. 48, 2477–2482. doi: 10.1128/AAC.48.7.2477-2482.2004 15215097 PMC434204

[B10] Chen T. Jackson J. W. Tams R. N. Davis S. E. Sparer T. E. Reynolds T. B. (2019a). Exposure of Candida albicans β (1,3)-glucan is promoted by activation of the Cek1 pathway. PLoS Genet. 15, e1007892. doi: 10.1371/journal.pgen.1007892 30703081 PMC6372213

[B11] Chen T. Wagner A. S. Reynolds T. B. (2022). When Is It Appropriate to Take Off the Mask? Signaling Pathways That Regulate ß(1,3)-Glucan Exposure in &lt;i<Candida albicans&lt;/i<. Front. Fungal Biol. 3. doi: 10.3389/ffunb.2022.842501 PMC10003681 36908584

[B12] Chen T. Wagner A. S. Tams R. N. Eyer J. E. Kauffman S. J. Gann E. R.. (2019b). Lrg1 Regulates β (1,3)-Glucan Masking in Candida albicans through the Cek1 MAP Kinase Pathway. MBio 10 (5), e01767–19. doi: 10.1128/mBio.01767-19 31530671 PMC6751057

[B13] Chen S. Zhou Y. Chen Y. Gu J. (2018). fastp: an ultra-fast all-in-one FASTQ preprocessor. Bioinformatics 34, i884–i890. doi: 10.1093/bioinformatics/bty560 30423086 PMC6129281

[B14] Correia I. Prieto D. Román E. Wilson D. Hube B. Alonso-Monge R.. (2019). Cooperative role of MAPK pathways in the interaction of candida albicans with the host epithelium. Microorganisms 8 (1), 48. doi: 10.3390/microorganisms8010048 31881718 PMC7023383

[B15] Cortés J. C. G. Curto M.-Á. Carvalho V. S. D. Pérez P. Ribas J. C. (2019). The fungal cell wall as a target for the development of new antifungal therapies. Biotechnol. Adv. 37, 107352. doi: 10.1016/j.bioteChadv.2019.02.008 30797093

[B16] Cui X. Wang L. Lü Y. Yue C. (2022). Development and research progress of anti-drug resistant fungal drugs. J. Infect. Public Health 15, 986–1000. doi: 10.1016/j.jiph.2022.08.004 35981408

[B17] Curto M.Á. Butassi E. Ribas J. C. Svetaz L. A. Cortés J. C. G. (2021). Natural products targeting the synthesis of β(1,3)-D-glucan and chitin of the fungal cell wall. Existing drugs and recent findings. Phytomedicine 88, 153556. doi: 10.1016/j.phymed.2021.153556 33958276

[B18] d’Enfert C. Kaune A.-K. Alaban L.-R. Chakraborty S. Cole N. Delavy M.. (2021). The impact of the Fungus-Host-Microbiota interplay upon Candida albicans infections: current knowledge and new perspectives. FEMS Microbiol. Rev. 45 (3), fuaa060. doi: 10.1093/femsre/fuaa060 33232448 PMC8100220

[B19] Da W. Shao J. Li Q. Shi G. Wang T. Wu D.. (2019). Physical interaction of sodium houttuyfonate with β-1,3-glucan evokes candida albicans cell wall remodeling. Front. Microbiol. 10. doi: 10.3389/fmicb.2019.00034 PMC6357593 30740095

[B20] Elgammal Y. Salama E. A. Seleem M. N. (2024). Enhanced antifungal activity of posaconazole against Candida auris by HIV protease inhibitors, atazanavir and saquinavir. Sci. Rep. 14, 1571. doi: 10.1038/s41598-024-52012-8 38238403 PMC10796399

[B21] Fisher M. C. Alastruey-Izquierdo A. Berman J. Bicanic T. Bignell E. M. Bowyer P.. (2022). Tackling the emerging threat of antifungal resistance to human health. Nat. Rev. Microbiol. 20, 557–571. doi: 10.1038/s41579-022-00720-1 35352028 PMC8962932

[B22] Fonzi W. A. (1999). PHR1 and PHR2 of Candida albicans encode putative glycosidases required for proper cross-linking of beta-1,3- and beta-1,6-glucans. J. Bacteriol. 181, 7070–7079. doi: 10.1128/JB.181.22.7070-7079.1999 10559174 PMC94183

[B23] Galán-Díez M. Arana D. M. Serrano-Gómez D. Kremer L. Casasnovas J. M. Ortega M.. (2010). Candida albicans beta-glucan exposure is controlled by the fungal CEK1-mediated mitogen-activated protein kinase pathway that modulates immune responses triggered through dectin-1. Infect. Immun. 78, 1426–1436. doi: 10.1128/IAI.00989-09 20100861 PMC2849429

[B24] Gow N. A. R. Latge J.-P. Munro C. A. (2017). The fungal cell wall: structure, biosynthesis, and function. Microbiol. Spectr. 5 (3). doi: 10.1128/microbiolspec.FUNK-0035-2016 PMC11687499 28513415

[B25] Grigor’eva A. Bardasheva A. Tupitsyna A. Amirkhanov N. Tikunova N. Pyshnyi D.. (2020). Changes in the ultrastructure of candida albicans treated with cationic peptides. Microorganisms 8 (4), 582. doi: 10.3390/microorganisms8040582 32316565 PMC7232200

[B26] Guirao-Abad J. P. Sánchez-Fresneda R. MaChado F. Argüelles J. C. Martínez-Esparza M. (2018). Micafungin Enhances the Human Macrophage Response to Candida albicans through β-Glucan Exposure. Antimicrob. Agents Chemother. 62 (5), e02161–17. doi: 10.1128/AAC.02161-17 29483123 PMC5923102

[B27] Heard S. C. Wu G. Winter J. M. (2021). Antifungal natural products. Curr. Opin. Biotechnol. 69, 232–241. doi: 10.1016/j.copbio.2021.02.001 33640596

[B28] Hoehamer C. F. Cummings E. D. Hilliard G. M. Rogers P. D. (2010). Changes in the proteome of Candida albicans in response to azole, polyene, and echinocandin antifungal agents. Antimicrob. Agents Chemother. 54, 1655–1664. doi: 10.1128/AAC.00756-09 20145080 PMC2863685

[B29] Holtappels M. Swinnen E. De Groef L. Wuyts J. Moons L. Lagrou K.. (2017). Antifungal activity of oleylphosphocholine on *in vitro* and *in vivo* candida albicans biofilms. Antimicrob. Agents Chemother. 62 (1), e01767–17. doi: 10.1128/AAC.01767-17 29061737 PMC5740347

[B30] Hopke A. Brown A. J. P. Hall R. A. Wheeler R. T. (2018). Dynamic fungal cell wall architecture in stress adaptation and immune evasion. Trends Microbiol. 26, 284–295. doi: 10.1016/j.tim.2018.01.007 29452950 PMC5869159

[B31] Hopke A. Nicke N. Hidu E. E. Degani G. Popolo L. Wheeler R. T. (2016). Neutrophil attack triggers extracellular trap-dependent candida cell wall remodeling and altered immune recognition. PLoS Pathog. 12, e1005644. doi: 10.1371/journal.ppat.1005644 27223610 PMC4880299

[B32] Huang X. Liu Y. Ni T. Li L. Yan L. An M.. (2020). 11g, a potent antifungal candidate, enhances candida albicans immunogenicity by unmasking β-glucan in fungal cell wall. Front. Microbiol. 11. doi: 10.3389/fmicb.2020.01324 PMC7338940 32695076

[B33] Huang T. Liu Y. Zhang C. (2019). Pharmacokinetics and bioavailability enhancement of baicalin: A review. Eur. J. Drug Metab. Pharmacokinet. 44, 159–168. doi: 10.1007/s13318-018-0509-3 30209794

[B34] Huang X. Yi Y. Yong J. Sun J. Song Z. Li D.. (2021). Inhibitory effect of berberine hydrochloride against Candida albicans and the role of the HOG-MAPK pathway. J. Antibiot. (Tokyo). 74, 807–816. doi: 10.1038/s41429-021-00463-w 34408288

[B35] Ibe C. Munro C. A. (2021). Fungal cell wall: An underexploited target for antifungal therapies. PLoS Pathog. 17, e1009470. doi: 10.1371/journal.ppat.1009470 33886695 PMC8061829

[B36] Jin X. Zhang M. Lu J. Duan X. Chen J. Liu Y.. (2021). Hinokitiol chelates intracellular iron to retard fungal growth by disturbing mitochondrial respiration. J. Adv. Res. 34, 65–77. doi: 10.1016/j.jare.2021.06.016 35024181 PMC8655124

[B37] Kim D. Langmead B. Salzberg S. L. (2015). HISAT: a fast spliced aligner with low memory requirements. Nat. Methods 12, 357–360. doi: 10.1038/nmeth.3317 25751142 PMC4655817

[B38] Lee K. K. Maccallum D. M. Jacobsen M. D. Walker L. A. Odds F. C. Gow N. A. R.. (2012). Elevated cell wall chitin in Candida albicans confers echinocandin resistance in *vivo* . Antimicrob. Agents Chemother. 56, 208–217. doi: 10.1128/AAC.00683-11 21986821 PMC3256049

[B39] Lee Y. Puumala E. Robbins N. Cowen L. E. (2021). Antifungal drug resistance: molecular mechanisms in candida albicans and beyond. Chem. Rev. 121, 3390–3411. doi: 10.1021/acs.chemrev.0c00199 32441527 PMC8519031

[B40] Li B. Dewey C. N. (2011). RSEM: accurate transcript quantification from RNA-Seq data with or without a reference genome. BMC Bioinf. 12, 323. doi: 10.1186/1471-2105-12-323 PMC3163565 21816040

[B41] Lima S. L. Colombo A. L. de Almeida Junior J. N. (2019). Fungal cell wall: emerging antifungals and drug resistance. Front. Microbiol. 10. doi: 10.3389/fmicb.2019.02573 PMC6881460 31824443

[B42] Liu X. Ma Z. Zhang J. Yang L. (2017). Antifungal compounds against candida infections from traditional chinese medicine. BioMed. Res. Int. 2017, 4614183. doi: 10.1155/2017/4614183 29445739 PMC5763084

[B43] Lopes J. P. Lionakis M. S. (2022). Pathogenesis and virulence of Candida albicans. Virulence 13, 89–121. doi: 10.1080/21505594.2021.2019950 34964702 PMC9728475

[B44] Love M. I. Huber W. Anders S. (2014). Moderated estimation of fold change and dispersion for RNA-seq data with DESeq2. Genome Biol. 15, 550. doi: 10.1186/s13059-014-0550-8 25516281 PMC4302049

[B45] McLellan C. A. Whitesell L. King O. D. Lancaster A. K. Mazitschek R. Lindquist S. (2012). Inhibiting GPI anchor biosynthesis in fungi stresses the endoplasmic reticulum and enhances immunogenicity. ACS Chem. Biol. 7, 1520–1528. doi: 10.1021/cb300235m 22724584

[B46] Mio T. Yabe T. Sudoh M. Satoh Y. Nakajima T. Arisawa M.. (1996). Role of three chitin synthase genes in the growth of Candida albicans. J. Bacteriol. 178, 2416–2419. doi: 10.1128/jb.178.8.2416-2419.1996 8636047 PMC177954

[B47] Montoya M. C. Beattie S. Alden K. M. Krysan D. J. (2020). Derivatives of the antimalarial drug mefloquine are broad-spectrum antifungal molecules with activity against drug-resistant clinical isolates. Antimicrob. Agents Chemother. 64 (3), e02331–19. doi: 10.1128/AAC.02331-19 31907188 PMC7038245

[B48] Pertea M. Pertea G. M. Antonescu C. M. Chang T.-C. Mendell J. T. Salzberg S. L. (2015). StringTie enables improved reconstruction of a transcriptome from RNA-seq reads. Nat. Biotechnol. 33, 290–295. doi: 10.1038/nbt.3122 25690850 PMC4643835

[B49] Pradhan A. Avelar G. M. Bain J. M. Childers D. Pelletier C. Larcombe D. E.. (2019). Non-canonical signalling mediates changes in fungal cell wall PAMPs that drive immune evasion. Nat. Commun. 10, 5315. doi: 10.1038/s41467-019-13298-9 31757950 PMC6876565

[B50] Pristov K. E. Ghannoum M. A. (2019). Resistance of Candida to azoles and echinocandins worldwide. Clin. Microbiol. Infect. Off. Publ. Eur. Soc Clin. Microbiol. Infect. Dis. 25, 792–798. doi: 10.1016/j.cmi.2019.03.028 30965100

[B51] Roy M. Karhana S. Shamsuzzaman M. Khan M. A. (2023). Recent drug development and treatments for fungal infections. Braz. J. Microbiol. 54, 1695–1716. doi: 10.1007/s42770-023-00999-z 37219748 PMC10484882

[B52] Sherrington S. L. Sorsby E. Mahtey N. Kumwenda P. Lenardon M. D. Brown I.. (2017). Adaptation of Candida albicans to environmental pH induces cell wall remodelling and enhances innate immune recognition. PLoS Pathog. 13, e1006403. doi: 10.1371/journal.ppat.1006403 28542528 PMC5456412

[B53] Shields R. K. Kline E. G. Healey K. R. Kordalewska M. Perlin D. S. Nguyen M. H.. (2018). Spontaneous Mutational Frequency and FKS Mutation Rates Vary by Echinocandin Agent against Candida glabrata. Antimicrob. Agents Chemother. 63 (1), e01692–18. doi: 10.1128/AAC.01692-18 30373796 PMC6325211

[B54] Tripathi A. Liverani E. Tsygankov A. Y. Puri S. (2020). Iron alters the cell wall composition and intracellular lactate to affect Candida albicans susceptibility to antifungals and host immune response. J. Biol. Chem. 295, 10032–10044. doi: 10.1074/jbc.RA120.013413 32503842 PMC7380197

[B55] Trovato L. Bongiorno D. Calvo M. Migliorisi G. Boraccino A. Musso N.. (2021). Resistance to echinocandins complicates a case of candida albicans bloodstream infection: A case report. J. Fungi (Basel Switzerland) 7 (6), 405. doi: 10.3390/jof7060405 PMC8224343 34064200

[B56] Wagner A. S. Lumsdaine S. W. Mangrum M. M. Reynolds T. B. (2023). Caspofungin-induced β(1,3)-glucan exposure in Candida albicans is driven by increased chitin levels. MBio 14, e0007423. doi: 10.1128/mbio.00074-23 37377417 PMC10470516

[B57] Walker L. A. Munro C. A. (2020). Caspofungin induced cell wall changes of candida species influences macrophage interactions. Front. Cell. Infect. Microbiol. 10. doi: 10.3389/fcimb.2020.00164 PMC7247809 32528900

[B58] Walker L. A. Munro C. A. de Bruijn I. Lenardon M. D. McKinnon A. Gow N. A. R. (2008). Stimulation of chitin synthesis rescues Candida albicans from echinocandins. PLoS Pathog. 4, e1000040. doi: 10.1371/journal.ppat.1000040 18389063 PMC2271054

[B59] Wang T. Shao J. Da W. Li Q. Shi G. Wu D.. (2018). Strong synergism of palmatine and fluconazole/itraconazole against planktonic and biofilm cells of candida species and efflux-associated antifungal mechanism. Front. Microbiol. 9. doi: 10.3389/fmicb.2018.02892 PMC6287112 30559726

[B60] Wang T. Shi G. Shao J. Wu D. Yan Y. Zhang M.. (2015). *In vitro* antifungal activity of baicalin against Candida albicans biofilms via apoptotic induction. Microb. Pathog. 87, 21–29. doi: 10.1016/j.micpath.2015.07.006 26169236

[B61] Wang Q. Wang Z. Xu C. Wu D. Wang T. Wang C.. (2024). Physical impediment to sodium houttuyfonate conversely reinforces β-glucan exposure stimulated innate immune response to Candida albicans. Med. Mycol. 62 (3), myae014. doi: 10.1093/mmy/myae014 38389246

[B62] Xie Y. Hua H. Zhou P. (2022). Magnolol as a potent antifungal agent inhibits Candida albicans virulence factors via the PKC and Cek1 MAPK signaling pathways. Front. Cell. Infect. Microbiol. 12. doi: 10.3389/fcimb.2022.935322 PMC9355038 35937692

[B63] Yang S. Fu Y. Wu X. Zhou Z. Xu J. Zeng X.. (2014). Baicalin prevents Candida albicans infections via increasing its apoptosis rate. Biochem. Biophys. Res. Commun. 451, 36–41. doi: 10.1016/j.bbrc.2014.07.040 25065741

[B64] Yang M. Solis N. V. Marshall M. Garleb R. Zhou T. Wang D.. (2022). Control of β-glucan exposure by the endo-1,3-glucanase Eng1 in Candida albicans modulates virulence. PLoS Pathog. 18, e1010192. doi: 10.1371/journal.ppat.1010192 34995333 PMC8775328

