# Supplementary figures and images for: Baicalin promotes β-1,3-glucan exposure in Candida albicans and enhances macrophage response
Source: Front Cell Infect Microbiol. 2024 Dec 9;14:1487173. doi: 10.3389/fcimb.2024.1487173 (PMC11664218; doi:10.3389/fcimb.2024.1487173)

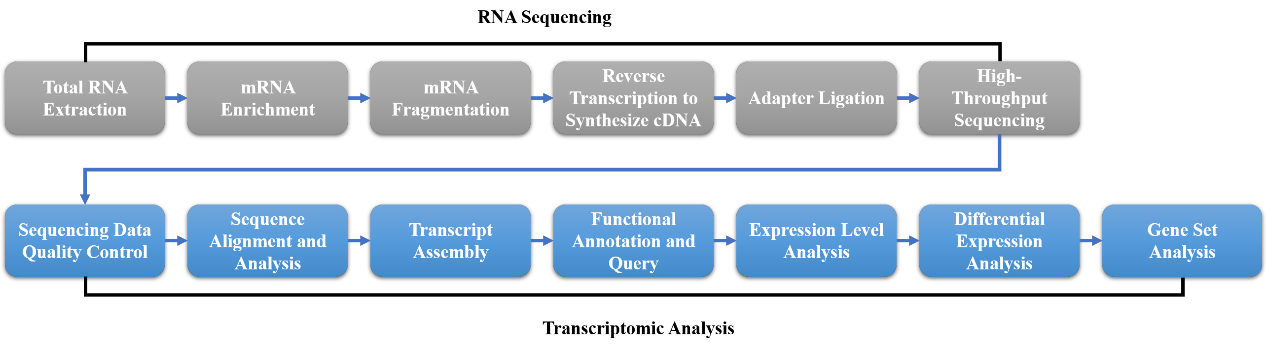


**Supplementary Figure S1.** Workflow of RNA sequencing and transcriptomic analysis.

Supplement: Supplementary file 2 [file Table1.docx]
